# Chemical Composition, Antimicrobial and Antioxidant Activities of Essential Oils from Organically Cultivated Fennel Cultivars

**DOI:** 10.3390/molecules16021366

**Published:** 2011-02-01

**Authors:** Abdelaaty A. Shahat, Abeer Y. Ibrahim, Saber F. Hendawy, Elsayed A. Omer, Faiza M. Hammouda, Fawzia H. Abdel-Rahman, Mahmoud A. Saleh

**Affiliations:** 1 Department of Phytochemistry, Production of Medicinal and Aromatic Plants, National Research Centre (NRC), 12311 Dokki, Cairo, Egypt; 2 Department of Cultivation, Production of Medicinal and Aromatic Plants, National Research Centre (NRC), 12311 Dokki, Cairo, Egypt; 3 Department of Biology, Texas Southern University, 3100 Cleburne Ave, Houston, TX 77004, USA; 4 Department of Chemistry, Texas Southern University, 3100 Cleburne Ave, Houston, TX 77004, USA

**Keywords:** *Foeniculum vulgare* var. *azoricum*, *Foeniculum vulgare* var. *dulce*, *Foeniculum vulgare* var. *vulgare*, antibacterial, antifungal, antioxidant, organic agriculture, GCMS, DPPH

## Abstract

Essential oils of the fruits of three organically grown cultivars of Egyptian fennel (*Foeniculum vulgare* var. *azoricum*, *Foeniculum vulgare* var. *dulce* and *Foeniculum vulgare* var. *vulgare*) were examined for their chemical constituents, antimicrobial and antioxidant activities. Gas chromatography/mass spectrometry analysis of the essential oils revealed the presence of 18 major monoterpenoids in all three cultivars but their percentage in each oil were greatly different. *trans-*Anethole, estragole, fenchone and limonene were highly abundant in all of the examined oils. Antioxidant activities of the essential oils were evaluated using the DPPH radical scavenging, lipid peroxidation and metal chelating assays. Essential oils from the *azoricum* and *dulce* cultivars were more effective antioxidants than that from the *vulgare* cultivar. Antimicrobial activities of each oil were measured against two species of fungi, two species of Gram negative and two species of Gram positive bacteria. All three cultivars showed similar antimicrobial activity.

## 1. Introduction 

*Foeniculum vulgare* Mill. is a biennial medicinal plant belonging to the family Apiaceae (Umbelliferae). Essential oil of fennel is used as flavoring agents in food products such as beverages, bread, pickles, pastries, and cheese. It is also used as a constituent of cosmetic and pharmaceutical products [1]. Herbal drugs and essential oils of fennel have hepatoprotective effects [2], as well as antispasmodic effects [3]. They are also known for their diuretic, anti-inflammatory, analgesic and antioxidant activities [4]. Anand *et al* [5] reported that fennel seed possesses anticancer activity. Recently it was shown that fennel essential oil possesses emmenagogue and galactagogue properties [6] and is a cure for pediatric colic and respiratory disorders due to its antispasmodic effects [7,8]. Many phytochemical studies have been conducted to investigate the chemical composition of the essential oil of fennel from different origins and have shown that the major components are phenylpropanoid derivatives and monoterpenoids [9,10,11]. Ethnobotanical data currently available on wild useful plants in Egypt highlight the importance of fennel’s culinary and medicinal uses [12]. Moreover, fennel has been used for centuries in the Mediterranean area as an aromatic herb and also in folk medicine, due to the aforementioned pharmacological properties of its essential oil.

Essential oil composition depends upon internal, environmental and agricultural practices as well as factors affecting the plant such as genetics, and ecological conditions [13,14]. According to Msaada *et al.* [15] maturation stages play an important factor influencing essential oil composition, while suitable environmental and agricultural practices would also help in improving yield and quality. Despite records on essential oil composition of fennel fruit [9,10,11], there are no phytochemical records on chemical composition variation of the three Egyptian fennel cultivars growing in organic farms without the use of pesticides or chemical fertilizers. This research was, therefore, conducted to comparatively examine the chemical composition, antioxidant and antimicrobial activity of the three organically grown fennel cultivars.

## 2. Results and Discussion

### 2.1 Chemical analysis

**Figure 1:**
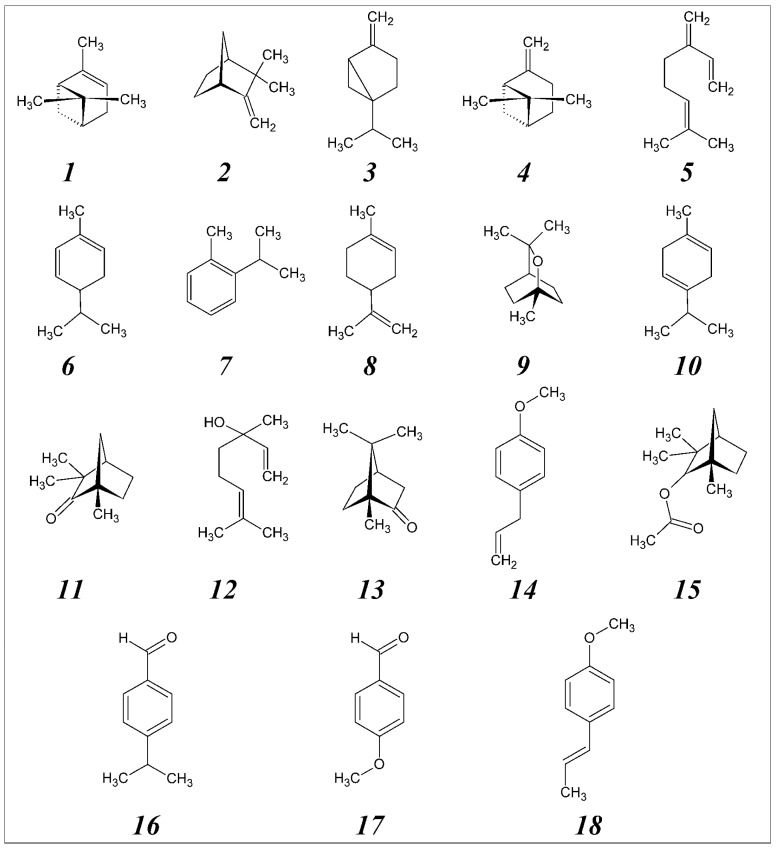
Chemical structures of the identified compounds from the essential oils with their ID # as presented in Table 1.

**Figure 2:**
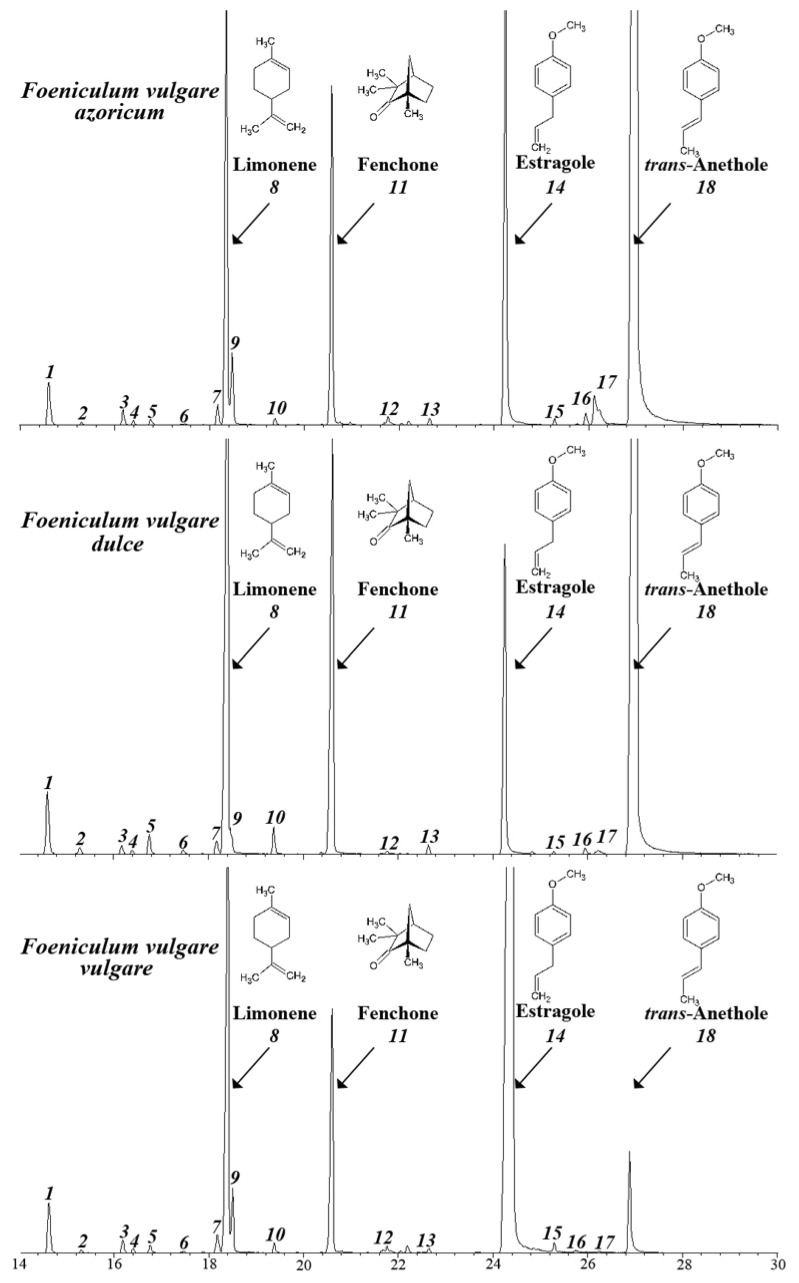
Total ion chromatograms of the three fennel cultivars essential oils.

Quantitative analyses of the chemical composition of the investigated essential oils of the three tested fennel cultivars are shown in Table 1. Gas chromatography/mass spectrometry (GCMS) analysis revealed the presence of 18 major chemicals in all three of the oils. Chemical identification of the oil constituents was conducted based on their retention time (t_R_), retention indices (KI) and mass spectral data, as well as by computer search of mass spectral databases. The chemical structures of the 18 identified compounds are shown in Figure 1. Total ion chromatograms of the three tested oils are shown in Figure 2.

It can be seen from both Table 1 and Figure 2 that the two *azoricum* and *dulce* cultivars are similar to some extent in their chemical composition but greatly different than the *vulgare* cultivar. *trans*-Anethole accounted for 61% and 46% in the oil of *azoricum* and *dulce* cultivars respectively, while it accounted for only 5% in the *vulgare* cultivar. On the other hand, estragole was the major compound in the oil of the *vulgare* cultivar, with a concentration of 58% compared to only 12% and 6% in the oils of *azoricum* and *dulce* cultivars, respectively. Other major components including pinene, limonene and fenchone were found in all of the three tested oils. Eucalyptol was detected in the *azoricum* and *vulgare* cultivars but was absent in the *dulce* cultivar. 

### 2.2. Antioxidant activity

Antioxidant activity of the three essential oils was evaluated by several complementary tests consisting of the DPPH free radical scavenging, the ferric reducing power (FRAP) assay, thiobarbituric acid reactive species assay (TBARS) and the ferrous ion-chelating (FIC) assay, using butylated hydroxytoluene (BHT) and ascorbic acid as references or positive controls. All of the assays were carried out at concentrations of 25, 50 and 100 mg/mL and results were reported as the average of three replicates. The concentrations that inhibited 50 % in each test (IC_50_ values) are shown in Table 2.

*Foeniculum vulgare* var. *azoricum* showed the highest activity in quenching of DPPH radical, even higher than either ascorbic acid or BHT. *Foeniculum vulgare* var. *dulce* showed compatible scavenging activity to ascorbic acid or BHT. The cultivar *Foeniculum vulgare* var. *vulgare* was the least effective radical scavenger, with an IC_50_ of 15.33 mg/mL which is about 44 and 37 times higher than the *azoricum* or *dulce* cultivars respectively.

Similar results were also obtained from the FRAP assay – both *azoricum* and *dulce* cultivars were similar in their antioxidant activity to ascorbic acid and BHT, however, the activity of the cultivar *vulgare* was much lower. In the TBARS assay the essential oil from the different *Foeniculum vulgare* cultivars showed inhibitory activity against lipid peroxidation with IC_50_ values of 0.08, 0.03 and 30.51 mg/mL, for the *azoricum, dulce* and *vulgare* cultivars, respectively (Table 2). *Foeniculum vulgare azoricum* at 100 mg/ mL showed approximately the same activity as BHT, but slightly higher than ascorbic acid in inhibition of lipid peroxidation. Formation of the Fe^2+^ complex did not reach completion in the presence of the fennel essential oils, indicating that essential oil chelates the iron. The metal chelating scavenging effect of fennel oils and standards decreased in the order of *azoricum* > *dulce* > BHA > ascorbic acid > *vulgare*. The data in Table 2 reveals that *azoricum* fennel has a remarkable capacity for iron binding, suggesting that is action as peroxidation protector may be related to its iron binding capacity. Based on the data obtained from this study, fennel essential oil exhibits ability as a free radical inhibitor or scavenging activity as well as primary antioxidant that reacts with free radicals, which may limit free radicals damage occurring in the human body.

### 2.3. Antimicrobial activity 

Antimicrobial activity of the essential oils obtained from the three fennel cultivars are shown in Table 3. Ampicillin was used as a reference material or positive control for the antibacterial activity and clotrimazole was used as a reference or positive control for the antifungal activity. The results indicated that all essential oil samples have antibacterial activity against Gram negative and Gram positive bacteria. The most effective oil against Gram negative bacteria was *Foeniculum vulgare azoricum*, which is less effective than ampicillin by 25% and 7% in the *Escherichia coli* and *Psedumonas aeruginosa* bioassays, respectively, while the most effective essential oil against Gram positive bacteria was *Foeniculum vulgare vulgare* which gave a larger inhibition zone than ampicillin by 58.3% and 114% in the *Staphylococcus aureus* and *Bacillus subtilis* tests, respectively. These data coincide with those of LoCantore *et al.* [16] who reported that fennel essential oil displayed a significant antibacterial activity, as determined with the agar diffusion method. The results also showed that *Foeniculum vulgare azoricum* was more effective antifungal than that of the reference commercial fungicidal clotrimazole. It produced 46% more inhibition (inhibition zone in mm) compared to the standard drug for *Aspergillus niger,* and it also has the same high activity against yeast, forming an inhibition zone larger than that of the standard drug by 40%. In conclusion, the essential oil of the three cultivars have antimicrobial activities and potent activity was observed with the *azoricum* variety in most cases, while *vulgare* cultivar is the most effective against Gram positive bacteria.

These recorded activities are in accordance with Khaldun [17], who reported that fennel oil had high antibacterial effect on *Candida albicans*, and bactericidal action on *Salmonella typhimurium* and *Salmonella dysenteriae*. Ozcan [18] also concluded that, the oils exerted varying levels of antifungal effects on the experimental mycelia growth of *Alternaria alternata*, *Fusarium oxysporum*, and *Rhizoctonia solani*.

## 3. Experimental 

### 3.1. Organic farming

Seeds of the three cultivars were obtained from the SEKEM Company of Egypt (3 Cairo-Belbeis Desert Road, El-Horreya, Cairo, Egypt). The soil was prepared for cultivation by adding 20 m^3^ compost, 200 Kg rock phosphate, 100 Kg feldspar to the soil on October 5, 2008 then irrigated to push weeds for growing up as well as plowing in rectangular way is required then establishing the rows via tractors. Seeds were manually sown on October 23, 2008 in lines which contain two irrigation dropping lines with 30 cm distance in between hoses. After sowing, seeds received a rich irrigation. The first process of weeding was carried out after the complete germination of seeds on November 4, 2008. Another process was done after thinning the seedlings to be two seedlings only in each hole (when the plant reached 8 cm in length). Four m^3^ of compost per feddan (4,200 m^2^) was added as organic fertilizer. The same kind of organic fertilizer was added at the rate of 4 m^3^/feddan during the third weeding. The first flowering was on February 02, 2009 and the harvest processes were conducted in May 23, 2009 by hands in the morning to avoid fruits escaping. Then moved to a clean drying location in the shade for seven to ten days for the separation of the fruits. The productivity per feddan was 800 Kg of fruits for *azoricum* and *dulce* and 1,200 kg for *vulgare*.

### 3.2. Isolation of essential oils

Fresh fennel fruits were dried at 35 °C in a drying cabinet and crushed to powders using a grinder. Essential oil was obtained by hydrodistillation of the powdered dry fruits according to the British Pharmacopeia Protocol [19]. The oil phase was separated, dried over anhydrous sodium sulfate, and kept in a dark glass bottle at 4 °C until the analyses.

### 3.3. Gas chromatography/mass spectrometry (GC/MS) analysis

GC/MS analysis of the essential oil was carried out using an HP5890 Series II Gas Chromatograph, HP 5972 Mass Selective Detector and Agilent 6890 Series Autosampler (Agilent Technologies, USA). A Supelco MDN-5S 30 m × 0.25 mm capillary column with a 0.5 μm film thickness was used with helium as the carrier gas at a flow rate of 1.0 mL/min. The GC oven temperature was programmed at an initial temperature of 40 °C for 5 minutes, then heated up to 140 °C at 5 °C /min and held at 140 °C for 5 min, then heated to 280 °C at 9 °C/min and held for five additional minutes. Injector and detector temperatures were set at 250 °C. Mass spectrometry was run in the electron impact mode (EI) at 70 eV. The identification of the chemical constituents of the oil was determined by their GC retention times, retention indices and interpretation of their mass spectra and confirmed by mass spectral library search using the National Institute of Standards and Technology (NIST) database with those of authentic samples or published data [20]. The retention indices were calculated for all of the volatile constituents using a homologous series of C_8_-C_20_ *n*-alkanes.

### 3.4. DPPH radical-scavenging activity

The antioxidant activity was determined using DPPH (1,1-diphenyl-2-picrylhydrazyl) radical scavenging model [21]. Three concentrations of tested essential oils (25, 50 and 100 µg/mL) prepared in methanol having a final DPPH radical concentration of 0.1mM. The mixture was shaken vigorously (2,500 rpm) for 1min then left to stand for 60 min in the dark. Scavenging capacity was measured spectrophotometrically at 517 nm. Ascorbic acid was used as a positive control. Inhibition (%) was plotted against the sample concentration in the reaction system. The percentage inhibition of the DPPH radical calculated according to the following formula:

% Inhibition = [(A _control_ −A _sample_) /A _control_] X 100

where A is absorbance.

### 3.5. Ferric reducing antioxidant power (FRAP)

The ferric reducing power of the essential oils was determined by using the potassium ferricyanide-ferric chloride method [11]. Different concentrations of three essential oils (25, 50 and 100 µg/mL) were added to 2.5 mL phosphate buffer (0.2M, pH 6.6) and 2.5 mL potassium ferricyanide (1%). The mixtures were incubated at 50 °C for 20 min, after which 2.5 mL trichloroacetic acid (10%) was added. An aliquot of the mixture (2.5 mL) was taken and mixed with 2.5 mL water and 0.5 mL 1% FeCl_3_. The absorbance at 700 nm was measured after allowing the solution to stand for 30 min. The FRAP of a sample is estimated in terms of Trolox equivalent antioxidant capacity (TEAC) in mM/L Trolox. Each assay was carried out in triplicate. Higher absorbance of the reaction mixture indicated greater reducing power.

### 3.6. Thiobarbituric acid reactive species test (TBARS)

The method of Daker *et al.* [22] was used to determine the thiobarbituric acid reactive substance (TBARS) [23], a secondary product of lipid peroxidation. For this, 0.1 mL of different dilutions of essential oils (25, 50 and 100 mg/mL) was added to mixture that contained 1 mL fowl egg yolk emulsified with 0.1 M phosphate buffer, pH 7.4, to obtain a final concentration of 25 g/L and 100 μL of 1 mM Fe^2+^. The mixture was incubated at 37 °C for 1 h, after which it was treated with 0.5 mL freshly prepared 15% trichloroacetic acid (TCA) and 1 mL 1% thiobarbituric acid (TBA). The reaction tubes were kept in a boiling water bath for 10 min. Upon cooling with ice, the tubes were centrifuged at 3,500 rpm for 10 min to remove precipitated protein. The formation of TBARS was measured by removing 100 μl supernatant and measuring the absorbance at 532 nm. The control was buffered egg yolk with Fe^2+^ only. Butylated hydroxyl toluene (BHT) and ascorbic acid were used as the standards. The percentage inhibition ratio was calculated from the following equation:

% Inhibition = [(A _control_ −A _sample_) /A _control_] X 100

where A = absorbance 

The concentration needed to achieve 50% inhibition (IC_50_) was determined by plotting the percentage of lipid peroxidation inhibition against essential oil concentrations. Each assay was carried out in triplicate.

### 3.7. Ferrous ion chelating ability assay

The ferrous ion-chelating (FIC) assay was carried out according to the method of Singh and Rajini [24]. Solutions of 2 mM FeCl_2_·4H_2_O and 5 mM ferrozine were diluted 20 times. Briefly, an aliquot (1 mL) of different concentrations of essential oils (25, 50 and 100 mg/mL) was mixed with 1 mL FeCl_2_·4H_2_O. After 5 min incubation, the reaction was initiated by the addition of ferrozine (1 mL). The mixture was shaken vigorously and after a further 10 min incubation period the absorbance of the solution was measured spectrophotometrically at 562 nm. The percentage inhibition of ferrous Fe^+2^ complex formation was calculated by using the formula:

% Inhibition = [(A _control_ −A _sample_) /A _control_] X 100

where A = absorbance

### 3.8. Antimicrobial activity

#### 3.8.1. Tested microorganisms

For the purpose of antimicrobial evaluation of the fennel cultivars essential oils, six microorganisms were used. Four bacterial species, *Pseudomonas aeruginosa* (Gram-negative), *Escherichia coli* (Gram-negative), *Staphylococcus aureus* (Gram-positive), *Bacillus subtilis* (Gram-positive), and two fungal species, *Aspergillus niger* and *Candida albicans* were employed for the determination of the antimicrobial activity. Bacteria and fungi were obtained from the stock cultures of Microbiology Laboratory at the National Research Centre in Giza, Egypt.

#### 3.8.2 Antimicrobial activity determination

To determine the antimicrobial activities of each tested essential oil, the disk diffusion method was utilized [25]. Using cultures of *Escerichia coli, Psedumonas aeruginosa, Staphylococcus aureus, Bacillus subtilis, Aspergillus niger, and Candida albicans*; bacterial species were cultured on nutrient agar media, while fungi were cultured on malt and yeast extract media. Inoculum suspension (10^4^ CFU mL^−1^) was prepared from each microorganism in broth media, nutrient broth inoculated with each bacterial species was incubated for 24 h at 37 °C; and malt and yeast extract broth with each fungal species were incubated for 48 h at 35 °C. Sterile filter paper disks (6.3 mm in diameter) were impregnated with 10 μL of each tested essential oil. The disks were allowed to dry at room temperature in a sterile airflow laminar chamber for one hour, then they were placed in the center of fresh nutrient agar plates or malt and yeast extract agar plates previously seeded with 100 μL of inoculum suspension of each bacterial and fungal species respectively. The cultures were incubated either at 35 °C for 72 h for filamentous fungus or at 37 °C for 24–48 h for yeasts and bacteria. Each experiment was replicated three times. Antibiotics were used as positive control; ampicillin was used as antibacterial standard, while miconazole nitrate was used as antifungal standard. The antimicrobial activities were evaluated by measuring the inhibition zone diameters (millimeters) surrounding each disk.

### 3.9. Statistical analysis

Conventional statistical methods were used to calculate means and standard deviations of three simultaneous assays carried out with the different methods. Analysis of variance (ANOVA) was applied to the data to determine differences (p < 0.05). To discover if there were significant differences between the levels of the main factor, contrasts (Tukey’s test) between means were made. [26] For the antioxidant activity, ANOVAs with two factors (essential oil and concentration) were applied for each parameter. The statistical analyses were made using Statgraphics 5.1 for Windows. A correlation between total phenols and antioxidant capacity was made using the function CORREL from Microsoft Excel software.

## 4. Conclusions

**Figure 3:**
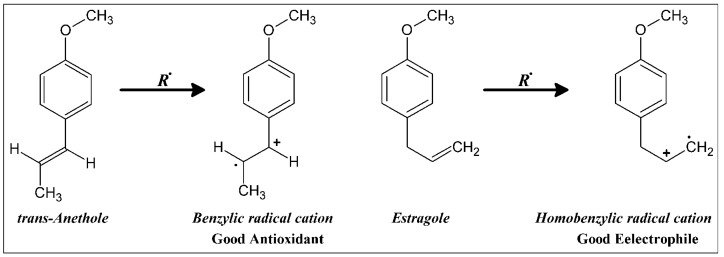
Radicals intermediates from anethole and estragole.

The essential oils of two of the fennel cultivars, *i.e. azoricum* and *dulce*, showed dramatically higher antioxidant activities than the essential oil of the *vulgare* cultivar. The two oils were similar in that *trans-*anethole was the major component in each oil, reaching 61% and 46% of the total oil of *azoricum* and *dulce*, respectively. On the other hand the essential oil of the *vulgare* cultivar contains only 5% *trans*-anethole and 58% estragole, which was only found at 12% and 6% in the other two cultivars. The fact that all of the three oils contain similar concentrations of all other major compounds excluding *trans*-anethole and estragole suggests that antioxidant activity is mostly related to the concentration of *trans*-anethole. One of the major differences between the chemical structure of estragole and anethole is the double bond of the propenyl side chain in anethole is conjugated with the aromatic ring while in estragole it is nonconjugated. *trans-*anethole can easily form a conjugated radical cation that can be easily delocalized with the aromatic ring and further stabilized by the methoxy group through the 1, 4 interaction in contrast to estragole which can only form homobenzyllic radical cation as shown in Figure 3. 

This difference between anethole and estragole was also observed in their free radical and photochemical dimerizations in which anethole dimerized by forming the intermediate radical cation but not estragole [27,28]. This observation may explain the differences in the antioxidant activity between the tested essential oils. It can also be concluded that anethole is a good radical scavenger but estragole is a good alkylation agent which might explain that estragole can easily alkylate DNA molecules and therefore, are suspected carcinogen as indicated by its ability to form covalent bonds with DNA bases [29].

Antimicrobial activities were similar among the three tested essential oils indicating that the antimicrobial effect of the oils is not related to radical interaction and both estragole and anethole are similar in their antimicrobial effects.

## Figures and Tables

**Table 1:** Chemical composition of the essential oils of fennel cultivars.

Name of Compound	CAS #	t_R_	KI	ID #	*Foeniculum vulgare*		
					*azoricum*	*dulce*	*vulgare*
α-Pinene	80-56-8	14.56	937	1	1.65	3.26	3.61
Camphene	79-92-5	15.25	953	2	0.08	0.30	0.19
Sabinene	3387-41-5	16.13	973	3	0.39	0.27	0.56
β-Pinene	127-91-3	16.35	981	4	0.12	0.14	0.21
Myrcene	123-35-3	16.72	991	5	0.18	0.66	0.32
α-Phellandrene	99-83-2	17.43	1005	6	0.11	0.18	0.11
o-Cymene	527-84-4	18.13	1020	7	0.46	0.38	0.71
Limonene	138-86-3	18.32	1034	8	12.53	27.78	20.64
Eucalyptol	470-82-6	18.44	1039	9	2.05	0.90	1.93
γ-Terpinene	99-85-4	19.35	1059	10	0.24	0.06	0.38
Fenchone	1195-79-5	20.54	1094	11	7.99	12.77	7.22
Linalool	598-07-2	21.73	1099	12	0.29	0.09	0.11
Camphor	76-22-2	22.60	1143	13	0.13	0.18	0.29
Estragole	140-67-0	24.21	1195	14	11.99	6.34	57.94
Fenchyl acetate	4057-31-2	25.25	1220	15	0.13	0.06	0.21
Cumic aldehyde	122-03-2	25.92	1224	16	0.18	0.06	0
*p*-Anisaldehyde	123-11-5	26.20	1252	17	0.40	0.11	0.26
*trans*-Anethole	4180-23-8	26.96	1283	18	61.11	46.26	4.99

**Table 2:** DPPH free radical scavenging effect of fennel essential oils.

Fennel cultivars essential oil and tested reference chemicals	IC_50_ (mg/mL)		
	DPPH	TBARS	Metal chelating
*azoricum*	0.35	0.08	2.23
*dulce*	0.41	0.03	2.51
*vulgare*	15.33	30.51	100.43
Ascorbic acid	0.40	5.89	117.11
BHT	0.44	0.002	45.15

**Table 3:** Antimicrobial activity of different fennel cultivars determined by disc diffusion assay.

Cultivars Oils and Reference Chemicals	Diameter of inhibition zone in mm					
	Bacteria				Fungi	
	Gram Negative		Gram Positive			
	*Escherichia coli*	*Pseudomonas aeruginosa*	*Staphylococcus aureus*	*Bacillus subtilis*	*Aspergillus niger*	*Candida albicans*
*azoricum*	15.0	13.0	14.0	15.0	19.0	21.0
*dulce*	9.0	12.0	15.0	13.0	18.0	16.0
*vulgare*	11.0	12.0	19.0	15.0	16.0	19.0
Ampicillin	20.0	14.0	12.0	7.0	-	-
Clotrimazole	-	-	-	-	13.0	15.0
